# Checklists to guide the supportive and critical care of tuberculous meningitis

**DOI:** 10.12688/wellcomeopenres.15512.2

**Published:** 2020-02-07

**Authors:** Joseph Donovan, Ursula K. Rohlwink, Elizabeth W. Tucker, Nguyen Thi Thu Hiep, Guy E. Thwaites, Anthony A. Figaji, Tuberculous Meningitis International Research Consortium, Rob E. Aarnoutse, Rob E. Aarnoutse, Suzanne T. B. Anderson, Suzanne T. B. Anderson, Nathan C. Bahr, Nguyen D. Bang, David R. Boulware, Tom Boyles, Lindsey H. M. te Brake, Satish Chandra, Felicia C. Chow, Fiona V. Cresswell, Reinout van Crevel, Angharad G. Davis, Sofiati Dian, Joseph Donovan, Kelly E. Dooley, Anthony Figaji, A. Rizal Ganiem, Ravindra Kumar Garg, Diana M. Gibb, Raph L. Hamers, Nguyen T. T. Hiep, Darma Imran, Akhmad Imron, Sanjay K. Jain, Sunil K. Jain, Byramee Jeejeebhoy, Jayantee Kalita, Rashmi Kumar, Vinod Kumar, Arjan van Laarhoven, Rachel P-J. Lai, Abi Manesh, Suzaan Marais, Vidya Mave, Graeme Meintjes, David B. Meya, Usha K. Misra, Manish Modi, Alvaro A. Ordonez, Nguyen H. Phu, Sunil Pradhan, Kameshwar Prasad, Alize M. Proust, Lalita Ramakrishnan, Ursula Rohlwink, Rovina Ruslami, Johannes F. Schoeman, James A. Seddon, Kusum Sharma, Omar Siddiqi, Regan S. Solomons, Nguyen T. T. Thuong, Guy E. Thwaites, Ronald van Toorn, Elizabeth W. Tucker, Sean A. Wasserman, Robert J. Wilkinson

**Affiliations:** 1 Oxford University Clinical Research Unit, Centre for Tropical Medicine, Ho Chi Minh City, Vietnam; 2 Centre for Tropical Medicine and Global Health, Nuffield Department of Medicine, University of Oxford, Oxford, UK; 3 Neuroscience Institute and Division of Neurosurgery, University of Cape Town, Cape Town, 7700, South Africa; 4 Department of Anesthesiology and Critical Care Medicine, Johns Hopkins University School of Medicine, Baltimore, MD, 21287, USA; 5 Division of Pediatric Critical Care, Johns Hopkins All Children’s Hospital, St. Petersburg, FL, USA; 6 Center for Tuberculosis Research, Johns Hopkins University School of Medicine, Baltimore, MD, 21287, USA

**Keywords:** Tuberculous meningitis, critical care, checklist, proforma

## Abstract

The assessment and management of tuberculous meningitis (TBM) is often complex, yet no standardised approach exists, and evidence for the clinical care of patients, including those with critical illness, is limited. The roles of proformas and checklists are increasing in medicine; proformas provide a framework for a thorough approach to patient care, whereas checklists offer a priority-based approach that may be applied to deteriorating patients in time-critical situations.

We aimed to develop a comprehensive assessment proforma and an accompanying ‘priorities’ checklist for patients with TBM, with the overriding goal being to improve patient outcomes. The proforma outlines what should be asked, checked, or tested at initial evaluation and daily inpatient review to assist supportive clinical care for patients, with an adapted list for patients in critical care. It is accompanied by a supporting document describing why these points are relevant to TBM. Our priorities checklist offers a useful and easy reminder of important issues to review during a time-critical period of acute patient deterioration. The benefit of these documents to patient outcomes would require investigation; however, we hope they will promote standardisation of patient assessment and care, particularly of critically unwell individuals, in whom morbidity and mortality remains unacceptably high.

## Disclaimer

The views expressed in this article are those of the authors. Publication in Wellcome Open Research does not imply endorsement by Wellcome.

## Abbreviations

AFB, acid-fast bacilli; ALT, alanine transaminase; ART, anti-retroviral therapy; AST, aspartate aminotransferase; BCG, Bacillus Calmette Guerin; CO _2_, carbon dioxide; CBF, cerebral blood flow; CPP, cerebral perfusion pressure; CRP, C-reactive protein; CSF, cerebrospinal fluid; CSW, cerebral salt wasting; CT, computed tomography; CVP, central venous pressure; ESR, erythrocyte sedimentation rate; ETCO _2_, end-tidal carbon dioxide; ETV, endoscopic third ventriculostomy; EVD, external ventricular drainage; GCS, Glasgow coma scale; HIV, human immunodeficiency virus; ICP, intracranial pressure; ICU, intensive care unit; INR, international normalised ratio; IVC, inferior vena cava; MAP, mean arterial pressure; MRI, magnetic resonance imaging; NIRS, near-infrared spectroscopy; ONSD, optic nerve sheath diameter; PbtO2, brain tissue oxygen tension monitoring; SIADH, syndrome of inappropriate diuretic hormone secretion; TB, tuberculosis; TBI, traumatic brain injury; TBM, tuberculous meningitis; TCD, transcranial Doppler; VP, ventriculoperitoneal

## Introduction

Tuberculous meningitis (TBM) is the most severe form of tuberculosis (TB) and causes critical illness from direct neurological injury or complications from the infection, treatment, or prolonged hospitalisation.

Due to serious complications, the management of TBM is complex, and includes supportive medical and neurosurgical measures. Currently there are no guidelines or standardised approach for the assessment and management of TBM. This is due to limited literature to develop an evidence-based approach, and because each TBM patient is unique. However, common themes exist, and a comprehensive proforma to guide patient assessment could be the first step towards standardised management.

Checklists can be powerful tools to focus attention ^1^ and their use in the medical field is growing ^2– 4^. We aimed to develop a comprehensive proforma for the assessment and management of TBM as well as a priorities checklist for the decompensating patient. The document cannot account for every scenario, but is designed to identify priorities; i.e. potentially reversible factors that contribute to morbidity and mortality. Local modifications to increase uptake and tailor use to suit local needs are encouraged. ****

Accompanying our proforma and checklist is the rationale for why these assessments may be important. Importantly, this article is not a guideline and does not make recommendations for care. It is not intended to replace a comprehensive ward round, nor to increase the clinical workload. Instead, it should provide a framework to highlight vital components during different stages of TBM care, with many complications overlapping throughout illness. We acknowledge that investigations and procedures will not be available at all centres.

## Comprehensive proforma

The comprehensive proforma is split into initial evaluation ( Table 1), daily inpatient review ( Table 2), and critical care in the intensive care unit (ICU) ( Table 3). However, as elements can occur at any time, the rationale is grouped by themes within sections titled “General supportive and critical care” and “Neurocritical care”.

**Table 1.** Initial evaluation.

Category	Specific question or assessment	✓
**History**		
	Age	
	Presenting complaints and duration (i.e., headache, irritability, vomiting, fever, neck stiffness, seizures, altered consciousness, lethargy, developmental regression, weight loss, night sweats, cough	
	Other respiratory symptoms	
	Previous treatment for tuberculosis	
	BCG immunisation	
	History of recent TB contact	
	Other previous illnesses or comorbidities	
	If HIV positive: - Date of diagnosis, treatment history, treatment adherence, recent CD4 and HIV viral load values	
**General clinical examination**		
	Weight and nutritional status	
	Vital signs (i.e., oxygen saturation, heart rate, blood pressure, temperature)	
	Hydration status (i.e., fluid input and output, clinical signs of dehydration)	
	Evidence of tuberculosis elsewhere (e.g., lung, lymph nodes)	
	BCG scar	
**Neurological examination**		
	Level of consciousness (i.e., GCS, modified for infants)	
	Pupillary exam (shape, size and reaction to light)	
	Assess for papilloedema by fundoscopy	
	Focal neurological deficits (i.e., cranial nerve palsies, hemiplegia, paraplegia, tetraplegia, urinary retention)	
	Head circumference and fontanelle in children	
**CSF examination (lumbar or ventricular)**		
	Opening pressure (immediately with needle insertion at lumbar puncture)	
	General appearance (i.e. colour, turbidity)	
**Laboratory tests (CSF)**		
	Lumbar or ventricular?	
	AFB smear	
	NAAT (e.g., GeneXpert)	
	Mycobacterial culture and drug susceptibility testing	
	White cell count (i.e., total and cell differential)	
	Protein	
	Glucose (paired with blood glucose)	
	Lactate	
**Laboratory tests (blood)**		
	Full blood count (i.e., haemoglobin, white blood cell count, platelets)	
	Non-specific inflammatory markers (i.e., ESR, CRP)	
	Electrolyte and renal function panel (i.e., sodium, potassium, glucose, creatinine, urea)	
	Liver function panel (i.e., ALT, AST, bilirubin)	
	Coagulation panel (i.e., INR, PTT)	
	HIV test (if positive, CD4 count and HIV viral load)	
	Serum osmolality (if hyponatraemia)	
**Laboratory tests (urine)**		
	Urine sodium (if hyponatraemia)	
	Urine osmolality (if hyponatraemia)	
**Imaging**		
	Chest X-ray	
	Brain and/or spine (i.e. CT or MRI)	
**Intracranial pressure measurement**		
	Intermittent measurements (i.e., lumbar puncture)	
	Continuous measurements (i.e., invasive monitoring with/ without drain)	
	Assessment for communicating hydrocephalus with air encephalogram or column test	
	Non-invasive estimates of ICP	

AFB, acid-fast bacilli; ALT, alanine transaminase; AST, aspartate aminotransferase; BCG, Bacillus Calmette Guerin; CRP, C-reactive protein; CSF, cerebrospinal fluid; CT, computed tomography; ESR, erythrocyte sedimentation rate; GCS, Glasgow coma scale; HIV, human immunodeficiency virus; INR, international normalised ratio; MRI, magnetic resonance imaging; NAAT, nucleic acid amplification test; PTT, prothrombin time; TB, tuberculosis. ‘✓’ can be selected when a proforma question has been answered, or a proforma point has been reviewed or tested.

**Table 2.** Daily inpatient review.

Category	Specific question or assessment	✓
**General clinical examination**		
	Weight and nutritional status (i.e., use of oral feeds, intravenous fluids, etc.)	
	Monitor for vomiting/inability to take drugs orally	
	Monitor for gastrointestinal bleeding	
	Vital signs (i.e., oxygen saturation, heart rate, blood pressure, temperature)	
	Hydration status (i.e., fluid input and output, clinical signs of dehydration, CVP, IVC ultrasound)	
**Medication evaluation**		
	Have any doses of anti-TB chemotherapy been missed?	
	Monitor for side effects from anti-TB chemotherapy	
	Check drug susceptibility testing results. Are changes to anti-TB chemotherapy required?	
	Monitor recent liver function and renal function panels for medication toxicity	
	Repeat liver function and renal function panels if toxicity concerns remain	
	Check corticosteroid dose	
	Schedule corticosteroid taper (i.e., when to reduce the dose)	
**Neurological examination**		
	Level of consciousness (i.e., GCS, modified for infants)	
	Assess for papilloedema by fundoscopy	
	Focal neurological deficits (i.e., cranial nerve palsies, hemiplegia, paraplegia, tetraplegia, urinary retention)	
	Has there been a change in examination since last review? If so, what is the suspected cause?	
	Does the patient need repeat neuroimaging?	
**Laboratory tests (blood)**		
	Repeat complete full blood count and inflammatory markers (i.e., if concern for other infection)	
	Repeat electrolyte and renal function panel (i.e., sodium, potassium, glucose, creatinine, urea) if change in hydration status	
	Repeat liver function panel (i.e., ALT, AST, bilirubin) if change in medications	
	Repeat serum osmolality if change in hydration status or new/worsening hyponatraemia	
**Laboratory tests (urine)**		
	Urine sodium if change in hydration status	
	Urine osmolality if change in hydration status	

ALT, alanine transaminase; AST, aspartate aminotransferase; CSF, cerebrospinal fluid; CVP, central venous pressure; GCS, Glasgow coma scale; IVC, inferior vena cava; TB, tuberculosis. ‘✓’ can be selected when a proforma question has been answered, or a proforma point has been reviewed or tested.

**Table 3.** Critical care.

Category	Specific question or assessment	✓
**General clinical examination**		
	Weight and nutritional status (i.e., use of oral feeds, intravenous fluids, etc.)	
	Monitor for vomiting/inability to take drugs orally	
	Monitor for gastrointestinal bleeding	
	Vital signs (i.e., oxygen saturation, heart rate, blood pressure, temperature)	
	Hydration status (i.e., fluid input and output, clinical signs of dehydration)	
	Monitor skin for pressure damage	
**Medication evaluation**		
	Have any doses of anti-TB chemotherapy been missed?	
	Monitor for side effects from anti-TB chemotherapy	
	Check drug susceptibility testing results. Are changes to anti-TB chemotherapy required?	
	Monitor recent liver function and renal function panels for medication toxicity	
	Repeat liver function and renal function panels if toxicity concerns remain	
	Check corticosteroid dose	
	Schedule corticosteroid taper (i.e., when to reduce the dose)	
**Vascular access**		
	Is central venous access still needed?	
	Is central venous access functioning properly?	
	Are there signs/symptoms of central line-associated blood stream infection?	
	Is invasive blood pressure monitoring (arterial line) still needed?	
**Urinary catheter**		
	Is the urinary catheter still needed?	
	Are there signs/symptoms of catheter-associated urinary tract infection?	
**Respiratory examination**		
	Monitor respiratory examination	
	Monitor ventilation with end tidal CO2 monitoring (if available)	
	Monitor ventilation and oxygenation with arterial blood gas sampling (if available)	
	Monitor and adjust mechanical ventilation settings/modes	
	Are there signs/symptoms of ventilator-associated pneumonia?	
	Repeat chest X-ray if ventilator-associated pneumonia suspected	
	Can removal of endotracheal tube be considered?	
**Neurological examination**		
	Follow up neurosurgical consultation (if applicable)	
	Level of consciousness (i.e., GCS, modified for infants) – is sedation required?	
	Has there been a change in examination since last review?	
	Assess for papilloedema by fundoscopy	
	Focal neurological deficits (i.e., cranial nerve palsies, hemiplegia, paraplegia, tetraplegia, urinary retention)	
	Is repeat neuroimaging needed?	
**Intracranial pressure optimisation**		
	Optimise head-of-bed elevation	
	Ensure appropriate sedation/analgesia	
	Check for fevers (if applicable, treat)	
	Check for appropriate blood pressure to determine cerebral perfusion pressure (if ICP is known)	
	Is continuous monitoring required (i.e., continuous parenchymal ICP or EVD)?	
	Consider non-invasive measures for evaluating ICP and brain perfusion	
**Post-operative neurosurgical management**		
	Monitor for changes in neurological exam	
	Monitor surgical wound for infection	
	Is repeat neuroimaging required (to check VP shunt or EVD placement)?	
	Plan for suture removal	
	If an EVD is *in situ*, check the level and check the drainage	
**Laboratory tests (blood)**		
	Repeat complete full blood count and inflammatory markers (i.e., if concern for other infection)	
	Repeat electrolyte and renal function panel (i.e., sodium, potassium, glucose, creatinine, urea) if change in hydration status	
	Repeat liver function panel (i.e., ALT, AST, bilirubin) if change in medications	
	Repeat serum osmolality if change in hydration status or new/worsening hyponatraemia	
**Laboratory tests (urine)**		
	Urine sodium if change in hydration status	
	Urine osmolality if change in hydration status	

ALT, Alanine transaminase; AST, aspartate aminotransferase; CBF, cerebral blood flow; EVD, external ventricular drain; GCS, Glasgow coma scale; ICP, intracranial pressure; TB, tuberculosis; VP, ventriculoperitoneal. ‘✓’ can be selected when a proforma question has been answered, or a proforma point has been reviewed or tested.

## General supportive and critical care

### History of present illness

Obtaining a thorough history of the patient’s signs and symptoms is paramount ( Table 1 and Box 1).

Box 1. **Key predictors of poor outcome in tuberculous meningitis**

Increased Medical Research Council TBM disease severity ^5– 8^

Reduced consciousness ^6, 9^

Hydrocephalus and raised ICP ^8, 10, 11^

Cerebral infarction ^6, 12^

Seizures ^12, 13^

HIV co-infection ^9, 14^

Multidrug resistant, or isoniazid mono-resistant, disease ^15^

Lower body weight ^5^

Younger and older age ^5, 16^

### General clinical examination and monitoring

General assessment and non-invasive monitoring of vital signs may be the only tools available to guide patient management in many centres. These provide valuable information in all care settings, and are mentioned in Table 1– Table 4.

**Table 4.** Priorities checklist for the acutely deteriorating patient with TBM.

	Reviewed
**Reduced consciousness**	
- Has the patient developed hydrocephalus, infarcts, cerebral venous thrombosis, or possible mass effect from tuberculomas, TB abscesses or IRIS? (consider repeat brain imaging [preferably with contrast], ICP monitoring)	
- Is the EVD or VP shunt working? (if applicable check EVD drainage, consider repeat imaging for VP shunt)	
- Is urgent neurosurgery required?	
- Have seizures been excluded?	
- Does serum glucose need correcting?	
- Does serum sodium need correcting?	
- Is there hypotension?	
**Systemically unwell**	
- Is supplemental oxygen required?	
- Are serum liver function tests elevated?	
- Do large urine outputs suggest hypovolaemia?	
- Is there gastrointestinal bleeding?	
- Are there signs of new infection?	

EVD, external ventricular drain; ICP, intracranial pressure; IRIS: immune reconstitution inflammatory syndrome; TBM, tuberculous meningitis; VP, ventriculoperitoneal. Check box for each checklist question when that question has been reviewed.

***Respiratory monitoring***. A change in neurological status may cause hypoxia due to airway compromise, and pulse oximetry may raise the alarm. Chest X-rays can help diagnose pulmonary TB, and in HIV co-infected patients with *Pneumocystis jirovecii*, pneumothorax, although this is rare ^17^.

***Heart rate monitoring***. Bradycardia may be caused by raised intracranial pressure (ICP) or brainstem ischaemia, and the development of tachycardia or bradycardia could indicate new infection or hypovolemia. ****

***Blood pressure monitoring***. Blood pressure monitoring may detect septic shock or cerebral salt wasting (CSW)-associated hypotension. It may also help calculate cerebral perfusion pressure (CPP).

***Temperature monitoring***. Fever is associated with worse outcomes in neurocritical care and an increased one-year mortality in HIV-uninfected individuals with TBM ^9^. Pyrexia may indicate superimposed bacterial infection.

### Medication evaluation and management

Important characteristics to monitor for anti-TB chemotherapy are described below and in Table 2.

***Anti-tuberculosis chemotherapy***. The optimum delivery of essential anti-TB chemotherapy is a priority, but optimal doses and administration routes are unknown ^18^. Prompt treatment and avoidance of therapy interruptions are essential to reduce mortality, and in unconscious patients, crushed medication administered via nasogastric tube, or intravenous therapy, may be considered ^18, 19^.

Anti-TB chemotherapy can change during the long duration of treatment. Regimen modifications may be necessitated by drug resistance, changes in weight, interactions with cytochrome P450-inhibiting anti-retroviral therapy (ART), and drug side effects, many of which cause and exacerbate critical illness ^18^. Rifampicin, isoniazid, pyrazinamide and fluoroquinolones can cause liver injury; therefore, regular monitoring of alanine transaminase (ALT), aspartate aminotransferase (AST), and bilirubin is important. Optimal management of drug-induced liver injury in TBM is unknown and is currently being studied ^20, 21^.

### Nutrition and the gastrointestinal tract

TB causes a chronic catabolic illness and patients commonly present with weight loss or failure to thrive in children. Additionally, TBM patients are at risk of the negative consequences of the catabolic stress of critical illness ^22^. Controlling glycaemia may be complicated by corticosteroids, which increase serum glucose. Nutrition in TBM often requires nasogastric tube placement when consciousness is reduced. Maintaining adequate nutrition is important to provide substrate for healing. Early feeding and avoidance of hypoglycaemia may improve outcomes after acute brain injury ^23^, but has not been studied in TBM.

Multidrug regimens, corticosteroids and anti-platelet drugs can lead to gastrointestinal intolerance and bleeding ^24^. Gastrointestinal bleeding may cause hypovolaemia and exacerbate reduced cerebral perfusion. Nausea and vomiting, common side effects of anti-TB chemotherapy, may further contribute to hypovolaemia. Aspiration of vomited gastric contents is a risk when consciousness is impaired. See Table 1 and Table 2.

### Kidney function, fluid balance, and electrolytes

***Kidney function***. Acute dehydration may place patients at risk for hypovolemia and prerenal acute kidney injury. Chronic use of anti-TB chemotherapy may be nephrotoxic ^25, 26^. Obtaining baseline kidney function tests, including creatinine and urea, may identify those at risk of acute or chronic kidney injury. Changes in urine output or fluid balance may precede laboratory abnormalities. See Table 1 and Table 2.

***Hyponatraemia and fluid balance***. Fluid balance is an important distinguishing parameter between the causes of hyponatremia (sodium <135mmol/L). Central venous pressure (CVP) reflects the intravascular volume and helps determine hydration status. CVP can be measured continuously or intermittently from a central venous catheter most often placed in the internal jugular vein or femoral vein if they are available. CSW is often characterised by high volume urine output, hypovolemia with low CVP, clinical signs of dehydration (dry mucous membranes, delayed capillary refill time, tachycardia, and hypotension) and haemoconcentrated laboratory parameters (elevated haematocrit, haemoglobin or urea) ^27^. Conversely, the syndrome of inappropriate anti-diuretic hormone (SIADH) lacks a high volume urine output, and patients are usually euvolaemic with a normal CVP, and no clinical signs of dehydration ^27^. Fluid balance charts may help identify these fluid shifts. Despite these distinguishing features, CSW and SIADH can be difficult to diagnose and other laboratory tests, such as serum and urinary osmolality and urinary sodium, may help further identify the aetiology, which is critical due to their divergent management approaches.

***Hypokalaemia***. Hypokalaemia may be a result of drugs or of poor nutrition, whereas hyperkalaemia may be due to hypoadrenalism either directly from TB or from withdrawal of corticosteroids. Monitoring low serum potassium and replacement requires an environment capable of close monitoring. The use of hypertonic saline, fludrocortisone and acetazolamide, individually or in combination, may rapidly shift electrolytes.

### Risks of prolonged critical care admission

Pressure ulcers are common in immobile individuals requiring prolonged care. Nosocomial infections occur due to changes in patients’ immune system and placement of foreign objects, such as a central venous line, arterial line, urinary catheter or endotracheal tube. Deep vein thrombosis is a risk of prolonged critical care admission. Urinary tract infections may occur secondary to urinary catheters. Further, impaired consciousness may require prolonged intubation and mechanical ventilation ^28^, which may be associated with a higher risk for gastrointestinal haemorrhage, septicaemia and pressures ulcers ^29^. Prolonged recovery and slow ventilator wean may involve tracheostomy placement.

## Neurocritical care

In addition to general and critical care management, specific neurocritical care may assist in TBM management.

### Neurological examination

***Level of consciousness***. The Glasgow coma scale (GCS) assesses level of consciousness. It can be confounded by intubation, sedatives, and pre-existing neurological conditions ^18, 30^. For paediatrics, modified GCS versions have been developed, but are used variably ^30^. Decreased GCS (<15) could be due to irreversible neurological injury and also reversible factors including electrolyte disturbances, raised ICP, seizures, and medication. Consequently, the GCS one week post-admission may be a stronger prognostic marker ^31^. A deteriorating GCS may signal worsening hydrocephalus, poorly controlled ICP, and progressive ischaemia.

***Focal neurological deficits***. Palsies commonly involve cranial nerves II, III and VI and can denote nerve arachnoiditis, ischaemia, a mass lesion, or hydrocephalus ^32^. Motor weakness and abnormalities may be due to ischaemia, infarction or brain shift.

***Cranial examination***. An enlarging head may signify subacute or chronic development of hydrocephalus in young children if their sutures are not fused.

### General monitoring in neurocritical care

***Blood pressure and cerebral perfusion***. Maintaining normotension is important for adequate CPP, defined as mean arterial pressure (MAP) minus ICP, which reflects the pressure gradient that drives cerebral blood flow (CBF) ^33^. Current treatment guidelines in traumatic brain injury (TBI) recommend maintaining age-appropriate CPP and MAP ^33– 36^. Hydration status and overall fluid balance, often compromised by CSW or poor oral intake, can affect MAP and subsequently CPP. TBM-specific goals for CPP and MAP are not established.

***Ventilation and oxygenation***. Oxygen administration may increase cerebral oxygenation and possibly reverse brain hypoxia ^37^. Oxygen and carbon dioxide (CO _2_) levels also affect CBF and ICP. Decreased arterial oxygen and increased CO _2_ both dilate cerebral vessels, which may increase cerebral blood volume and ICP ^38^. Conversely, aggressive hyperventilation may constrict cerebral vessels and cause ischaemia from decreased CBF ^38^. Therefore, tight control of end tidal carbon dioxide (ETCO _2_) is crucial to control raised ICP but avoid ischaemia and is an important parameter in TBI management guidelines, although the target level for TBM in unknown ^34, 39^. Both oxygenation and ventilation can be monitored noninvasively with pulse oximetry and ETCO _2_ or invasively with arterial blood gases.

***Temperature***. Hyperthermia may increase cerebral metabolic rate and CBF, which can further increase ICP in a swollen brain ^40^. Induced hypothermia is experimentally neuroprotective but is associated with poorer outcomes in TBI ^34, 35, 39^. The target temperature in TBM is not established.

***Head-of-bed elevation***. Elevating the head end of a bed lowers ICP through improved venous drainage and extracranial shift of cerebrospinal fluid (CSF). However, the MAP may also fall. In non-TBM pathology, studies suggest a beneficial or non-detrimental role of head-of-bed elevation to 30° ^41– 43^.

### Neuroimaging

Infarcts may not be visible on admission computed tomography (CT) scans ^44, 45^, but diffusion-weighted magnetic resonance imaging (MRI) is sensitive to acute/evolving infarcts ^46, 47^. Follow-up imaging is important to detect new infarcts ^44, 45, 48^ and, although there are little data on the temporal profile of infarct development, patients appear to be at greatest risk during the acute phase (first month).

Hydrocephalus is the commonest cause of increased ICP in TBM ^45, 49^. The communicating nature of hydrocephalus has implications for the safety of lumbar punctures and the method of treatment. However, communication cannot be determined from standard imaging ^50^ and lumbar exudate can confound determination of the level of CSF obstruction. ICP cannot be estimated from ventricular size alone on imaging ^48^. Hydrocephalus may persist or develop *de novo* over the first six months after treatment initiation; therefore, repeat neuroimaging may be warranted ^10, 51^.

Tuberculomas and TB abscesses occasionally complicate the management of TBM if they cause local mass effect ^52^ and precipitate CSF obstruction, focal deficits or seizures ^45, 48, 53, 54^. During treatment, paradoxical enlargement of established tuberculomas or development of new tuberculomas may necessitate follow-up imaging ^45, 53, 55– 57^.

Repeat neuroimaging can be used to monitor disease progression, evaluate deteriorating patients, and confirm the placement of a ventriculoperitoneal (VP) shunt or an external ventricular drain (EVD). The ideal timing of follow up imaging is unclear in stable patients.

### Raised intracranial pressure

Raised ICP is a key factor precipitating adverse outcomes. Firstly, it reduces CPP and exacerbates existing cerebral ischaemia due to vasculitis. Secondly, generalised or compartmentalised increased ICP causes brain shift and consequent neural injury. ICP monitoring is useful; non-invasive monitoring techniques may be used when gold-standard invasive monitoring is unavailable. New monitoring techniques have potential for improving patient care, yet these are not widely available. Important points for the monitoring and optimisation of ICP are shown in Table 3 and Table 4.

***Non-invasive intracranial pressure measurement and monitoring***

**Fundoscopy**

Optic disc swelling can indicate raised ICP. However, fundoscopy is challenging to perform and highly operator dependent, and the development of papilloedema can be delayed.

**Optic nerve sheath diameter ultrasound**

Changes in optic nerve sheath diameter (ONSD) due to raised ICP occur rapidly and can be measured using ultrasound ^58^. ONS ultrasound is quick, easy, and reproducible, and correlates with ICP ^59, 60^, although evidence for its use in TBM is limited ^61– 63^.

**Compromised cerebral perfusion**

Various neuromonitors have been used in non-TBM pathologies, to detect ischaemic brain injury ^64– 66^. These measure various facets of brain perfusion and each have strengths and limitations.

**Transcranial Doppler ultrasound**

Transcranial Doppler (TCD) can be used to measure flow velocity in basal vessels and detect vasculopathy; however, it may not detect mild-moderate ICP changes, is limited to flow in the major cerebral vessels, and is technically challenging ^18, 67^.

***Non-invasive cerebral oxygenation monitoring: near-infrared spectroscopy***. Near-infrared spectroscopy (NIRS) is a non-invasive monitor that uses optical technology to continuously assess brain oxygenation ^68, 69^. NIRS is limited by superficial penetration of ** cortex, distortion by the skull, CSF and oedema ^68, 70, 71^ and poor long term monitoring.

***Invasive cerebral oxygenation monitoring: partial pressure of brain tissue oxygen tension***. The partial pressure of brain tissue oxygen tension (PbtO2) monitor is a thin parenchymal catheter that offers continuous monitoring of brain oxygenation ^66, 72^. Normal values have not been established; however, the risk of poor outcome increases with PbtO2 <20mmHg ^73^, especially <10mmHg ^64, 72, 74, 75^.

***Invasive intracranial pressure measurement and monitoring***. CSF opening pressure may be measured from the ventricles with an EVD or via lumbar puncture (when safe) ^76^. CSF drainage allows simultaneous ICP monitoring and treatment. Continuous monitoring is possible with a parenchymal probe.

***Post-operative neurosurgery management***. This includes wound review, suture removal, and clinical monitoring for signs of treatment failure. Repeat imaging can check treatment success. EVDs must be carefully managed to avoid life-threatening complications (infection and overdrainage-related intracranial haemorrhage). VP shunts are permanent and therefore complication rates must be viewed over the full lifetime of the patient.

### Management of acutely decompensating patients

Causes for acute neurological decompensation include raised ICP, metabolic disturbances (i.e., hyponatremia, hypoglycaemia), stroke (ischaemic or haemorrhagic) and seizures. Table 4 outlines a priorities-based checklist approach to the acutely decompensating patient. The rationale for this checklist is described below, unless already discussed.

**Seizures**

Clinical and subclinical seizures can increase ICP due to the increased cerebral metabolic demand and resultant increased CBF. Hydrocephalus, infarcts, tuberculomas, and electrolyte imbalance can all precipitate seizures. Anti-convulsants that induce cytochrome P450 enzymes, or are susceptible to enzyme induction by rifampicin, may complicate management.

**Hyperosmolar treatment**

Intravenous administration of a hyperosmolar solution creates an osmotic gradient, removing water from the brain and decreasing ICP ^77^. Hypertonic saline may lower ICP faster, further, and for longer than mannitol ^18^; however, no trials have directly compared these agents in TBM.

***Raised intracranial pressure surgical management***

**Hydrocephalus**

VP shunting has long been standard practice for hydrocephalus but may be associated with complications ^78, 79^. EVD may be used for temporary drainage of CSF, and to assess the benefit of a VP shunt in patients with an altered sensorium. With endoscopic third ventriculostomy (ETV), CSF is drained internally by connecting the ventricles with the subarachnoid space via a stoma in the floor of the third ventricle. ETV is particularly challenging in TBM and experience is required ^80, 81^.

**Mass lesions**

Surgical excision for tuberculomas is uncommon but may be indicated depending on their size, location, expansion, and clinical consequences. Surgery is more commonly needed for TB abscesses (drainage and/or excision).

### Cerebral venous thrombosis

Cerebral venous thrombosis is an unusual cause of acute neurological deterioration in TBM, but has been described ^82, 83^.

## Data availability

No data are associated with this article.
