# Supplementary material for: Checklists to guide the supportive and critical care of tuberculous meningitis
Source: Wellcome Open Res. 2020 Feb 7;4:163. Originally published 2019 Oct 31. [Version 2] doi: 10.12688/wellcomeopenres.15512.2 (PMC6964359; doi:10.12688/wellcomeopenres.15512.2)
Supplement: Peer review history

# Peer review: Reviewer response for version 1

**DOI:** 10.21956/wellcomeopenres.16979.r37331

**Published:** 2020-01-15

**Authors:** Rakesh K. Gupta

**Affiliations:** 1 Department of Radiology and Imaging, Fortis Memorial Research Institute, Gurgaon, Haryana, India

The authors have attempted to streamline the management of TBM and its complications in a step wise fashion and is a welcome step. However, there is nothing new in what they have mentioned and is practised in the countries where the disease is endemic. I am fine with summary for management except for a small comment on table I and is as under:

 Table 1:

- Assessment for communicating hydrocephalus with air encephalogram or column test is obsolete and is only of historical relevance and should be deleted.

I confirm that I have read this submission and believe that I have an appropriate level of expertise to confirm that it is of an acceptable scientific standard.

# Response

**Published:** 2020-01-22

**Authors:** Joseph Donovan

**Affiliations:** Oxford University Clinical Research Unit, Ho Chi Min City, Vietnam

Dear Dr Rakesh K. Gupta,,

Thank you for reviewing our open letter “Checklists to guide the supportive and critical care of tuberculous meningitis”.

Please find below our point by point responses.

1. The authors have attempted to streamline the management of TBM and its complications in a step wise fashion and is a welcome step. However, there is nothing new in what they have mentioned and is practiced in the countries where the disease is endemic. I am fine with summary for management except for a small comment on table I and is as under:

Table 1: Assessment for communicating hydrocephalus with air encephalogram or column test is obsolete and is only of historical relevance and should be deleted.

Thank you for your comment. However, we disagree that this does not offer anything new; our proforma is the first comprehensive patient assessment tool for tuberculous meningitis. No other checklist exists to allow a priority-based approach to a deteriorating patient in this disease. Whilst the information included in the checklist and proforma may be accepted knowledge, or available elsewhere, our presentation of this information in proforma and checklist formats aim to support clinical assessment, and highlight vital components, during clinical care.

2. Table 1: Assessment for communicating hydrocephalus with air encephalogram or column test is obsolete and is only of historical relevance and should be deleted.

We note, but disagree with, the reviewer's comment on air encephalography and column tests. These are used as standard approaches in at least two big centres that publish on TB meningitis, based on published data, so this is hardly historical nor obsolete.

There is no current technology, apart from invasive methods, that have been shown to safely and reliably distinguish between communicating and non-communicating hydrocephalus. This is based on published studies. We know that some centres do not try to distinguish and therefore have higher rates of surgical procedures - VP shunting and endoscopy. However, with medical management, most patients can avoid those surgical procedures - this was published by Johan Schoeman many years ago and the results are as relevant today as they were then. But this of course depends on being able to do lumbar punctures safely, which may be risky for the 15-20% of patients that may have non-communicating hydrocephalus. To our knowledge, there has been no paper showing the safety and reliability of any imaging to confirm the communicating nature of hydrocephalus in TBM.

So the reviewer's comment is not evidence-based and we are comfortable that our manuscript reflects published data.

# Peer review: Reviewer response for version 1

**DOI:** 10.21956/wellcomeopenres.16979.r36903

**Published:** 2019-12-03

**Authors:** Abdu Kisekka Musubire

**Affiliations:** 1 Infectious Disease Institute, College of Health Sciences, Makerere University, Kampala, Uganda

The authors attempt to answer a very relevant question with scarce data. The article is timely and very important. I have the following concerns:

 Box 1 page 3: Define the extremes of ages for the readers

 Under respiratory monitoring: page 3

 Pneumothorax is rare in PJP

 Swiss Med Wkly. 2019 Oct 3;149:w20130. Imaging patterns of Pneumocystis jirovecii pneumonia in HIV-positive and renal transplant patients - a multicentre study. Christe A1, Walti L2, Charimo J1, Rauch A2, Furrer H2, Meyer A3, Huynh-Do U3, Heverhagen JT4, Mueller NJ5, Cavassini M6, Mombelli M6, van Delden C7, Frauenfelder T8, Montet X9, Beigelman-Aubry C10, Arampatzis S11, Ebner L3 ^1^.

 Indian J Crit Care Med. 2015 Jan;19(1):34-7. Paroxysmal sympathetic hyperactivity in neurological critical care. Verma R1, Giri P1, Rizvi I1 ^2^.

 Still on page 3, the roles for Heart rate monitoring are presented in a limited way 

 Tachycardia is common and directly associated with TBM.

 West Indian Med J. 2015 Dec;64(5):543-547. Epub 2016 Apr 29. Paroxysmal Sympathetic Hyperactivity in a Child with Tuberculous Meningitis A Case Study and Review of Related Literature. Xu Y1, Wan L2, Ning J2, Guo W2, Ren L2 ^3^.

 Int J Infect Dis. 2010 Jul;14(7):e586-91. Tuberculous meningitis in HIV-infected patients in Brazil: clinical and laboratory characteristics and factors associated with mortality.

 Croda MG1, Vidal JE, Hernández AV, Dal Molin T, Gualberto FA, de Oliveira AC ^4^.

 Author information

 Still under temperature monitoring. The reader is interested in clues as to how to differentiate the temperature of TBM from super imposed infection?

 Table 1 page 4: Relevance of the previous BCG scar?

 I propose that the tests be separated in to those with high utility like gene expert, low CSF glucose from those with low utility

 like AAFB, culture. Imaging must always be contrasted unless contraindicated

 Table 2: The part on monitoring for GIT bleeding, what is the practical way of doing this? Not so much information about its relevance in TBM patients.

 If possible the authors should expound on how to suspect superimposed infections 

 Table 4  page 7. In a rapidly deteriorating patient with TBM, paradoxical reactions like IRIS needs to be mentioned especially in HIV positive patients. And cerebral venous thrombosis(BMJ Case Rep. 2013 Aug 5;2013. pii: bcr2013009892 ^5^. Indian J Pediatr. 2019 Apr;86(4):371-378. Epub 2019 Jan 9 ^6^.

 Risks of prolonged hospital admission should include DVT.

 Page 9 Last paragraph under neuroimaging needs a reference.

I confirm that I have read this submission and believe that I have an appropriate level of expertise to confirm that it is of an acceptable scientific standard.

## References

[1] : Imaging patterns of Pneumocystis jirovecii pneumonia in HIV-positive and renal transplant patients – a multicentre study. *Swiss Medical Weekly*.2019; 10.4414/smw.2019.20130 10.4414/smw.2019.20130 31580472

[2] : Paroxysmal sympathetic hyperactivity in neurological critical care. *Indian Journal of Critical Care Medicine*.2015;19(1) : 10.4103/0972-5229.148638 34-37 10.4103/0972-5229.148638 PMC429640925624648

[3] : Paroxysmal Sympathetic Hyperactivity in a Child with Tuberculous Meningitis: A Case Study and Review of Related Literature. *West Indian Medical Journal*.2016; 10.7727/wimj.2016.055 10.7727/wimj.2016.055 PMC496134527398825

[4] : Tuberculous meningitis in HIV-infected patients in Brazil: clinical and laboratory characteristics and factors associated with mortality. *International Journal of Infectious Diseases*.2010;14(7) : 10.1016/j.ijid.2009.08.012 e586-e591 10.1016/j.ijid.2009.08.012 20005759

[5] : A rare presentation of cerebral venous sinus thrombosis associated with tubercular meningitis. *Case Reports*.2013;2013(aug05 1) : 10.1136/bcr-2013-009892 10.1136/bcr-2013-009892 PMC376166023917359

[6] : A Child with Tuberculous Meningitis Complicated by Cortical Venous and Cerebral Sino-Venous Thrombosis. *The Indian Journal of Pediatrics*.2019;86(4) : 10.1007/s12098-018-2830-x 371-378 10.1007/s12098-018-2830-x 30623313

# Response

**Published:** 2020-01-22

**Authors:** Joseph Donovan

**Affiliations:** Oxford University Clinical Research Unit, Ho Chi Min City, Vietnam

Dear Dr Abdu Kisekka Musubire,

Thank you for reviewing our open letter “Checklists to guide the supportive and critical care of tuberculous meningitis”.

Please find below our point by point responses.

1. Box 1 page 3: Define the extremes of ages for the readers

‘Older age’ is based upon a Cox regression model for 9-month survival in HIV uninfected adults with TBM. ^1^ An increase in age (per +10 years) gave a hazard ratio of 1.24 (95% CI 1.15-1.34, p <0.001). In this HIV uninfected group, median age was 40 years (IQR 27-56 years). Regarding ‘younger age’, in a study of 214 children with TBM (mean age at presentation 4.1 years), 49 patients (23%) died. ^2^ 63% of deaths were in age 5 or younger; however this age group is represented more frequently. Defining ‘extremes of age’ in adults is challenging; these are based on age ranges of individual studies. In paediatrics it is broadly accepted that most cases occur in children < 5yrs, and therefore more deaths are likely in that age range.

2. Under respiratory monitoring: page 3, Pneumothorax is rare in PJP

We have added here that this is rare and referenced this ^3^

3. Still on page 3, the roles for Heart rate monitoring are presented in a limited way

Tachycardia is common and directly associated with TBM.

Tachycardia is common with many medical conditions. In our article we have tried to focus on critical care of TBM. Whilst it clearly can occur, we do not feel paroxysmal sympathetic hyperactivity is sufficiently associated with TBM to include here. In the first cited study, ^4^ there was only one case of TBM associated with paroxysmal sympathetic hyperactivity. The second cited study ^5^ notes it is rare in TBM. We note the reference describing tachycardia being associated with mortality in a HIV infected cohort. ^6^

4. Still under temperature monitoring. The reader is interested in clues as to how to differentiate the temperature of TBM from super imposed infection?

This is a valid point, and we are not suggesting there are clues to differentiate these. We wish to note that fever in TBM may not only be due to TBM disease. Resolution of TBM-associated fever, followed by the development of new fever, may indicate new infection.

5. Table 1 page 4: Relevance of the previous BCG scar?

BCG vaccine protects against meningeal and miliary TB in infants ^7^

6. I propose that the tests be separated in to those with high utility like gene expert, low CSF glucose from those with low utility like AAFB, culture.

We feel the value of these tests will vary by site, and have therefore kept these listed together.

7. Imaging must always be contrasted unless contraindicated

This is a good point. However the addition of contrast may not always be possible. We have added ‘preferably with contrast’ to table 4

8. Table 2: The part on monitoring for GIT bleeding, what is the practical way of doing this? Not so much information about its relevance in TBM patients.

Recommending how to practically monitor for GI bleeding goes beyond the scope of this article. This adverse event is relevant given frequent dexamethasone use in TBM, and the increasing evidence base for aspirin ^8^

9. If possible the authors should expound on how to suspect superimposed infections

Although this is an important component to TBM care, we feel expanding on this is beyond the scope of this article

10. Table 4  page 7. In a rapidly deteriorating patient with TBM, paradoxical reactions like IRIS needs to be mentioned especially in HIV positive patients. And cerebral venous thrombosis(BMJ Case Rep. 2013 Aug 5;2013. pii: bcr2013009892 ^5^ . Indian J Pediatr. 2019 Apr;86(4):371-378. Epub 2019 Jan 9 ^6^ .

We have now specifically mentioned IRIS and cerebral sinus thrombosis in table 4.

In the supporting document we have added the sentence ‘Cerebral venous thrombosis is an unusual cause of acute neurological deterioration in TBM, but has been described’ with references.

11. Risks of prolonged hospital admission should include DVT.

We have added ‘Deep vein thrombosis is a risk of prolonged critical care admission’

12. Page 9 Last paragraph under neuroimaging needs a reference.

We have discussed the repeat imaging with references in the preceding paragraphs, and repeat imaging after placing hardware in the brain is standard neurosurgical practice

1            Thao LTP, Heemskerk AD, Geskus RB, *et al.* Prognostic Models for 9-Month Mortality in Tuberculous Meningitis. *Clin Infect Dis* 2018; **66**: 523–32.

2            Yaramiş A, Gurkan F, Elevli M, *et al.* Central nervous system tuberculosis in children: a review of 214 cases. *Pediatrics* 1998; **102**: E49.

3            Christe A, Walti L, Charimo J, *et al.* Imaging patterns of Pneumocystis jirovecii pneumonia in HIV-positive and renal transplant patients - a multicentre study. *Swiss Med Wkly* 2019; **149**: w20130.

4            Verma R, Giri P, Rizvi I. Paroxysmal sympathetic hyperactivity in neurological critical care. *Indian J Crit Care Med* 2015; **19**: 34–7.

5            Xu Y, Wan L, Ning J, Guo W, Ren L. Paroxysmal Sympathetic Hyperactivity in a Child with Tuberculous Meningitis A Case Study and Review of Related Literature. *West Indian Med J* 2015; **64**: 543–7.

6            Croda MG, Vidal JE, Hernández A V, Dal Molin T, Gualberto FA, de Oliveira ACP. Tuberculous meningitis in HIV-infected patients in Brazil: clinical and laboratory characteristics and factors associated with mortality. *Int J Infect Dis* 2010; **14**: e586-91.

7            Mangtani P, Abubakar I, Ariti C, *et al.* Protection by BCG vaccine against tuberculosis: A systematic review of randomized controlled trials. *Clin Infect Dis* 2014; **58**: 470–80.

8            Mai NT, Dobbs N, Phu NH, *et al.* A randomised double blind placebo controlled phase 2 trial of adjunctive aspirin for tuberculous meningitis in HIV-uninfected adults. *Elife* 2018; **7**. DOI:10.7554/eLife.33478.
